# The effect of using the minimized cardio-pulmonary bypass Systems for Coronary Artery Bypass Grafting in diabetic patients

**DOI:** 10.1186/s13019-021-01551-6

**Published:** 2021-06-07

**Authors:** Turki B. Albacker, Mohammed Fouda, Bakir M. Bakir, Ahmed Eldemerdash

**Affiliations:** Cardiac Sciences Department, College of Medicine, King Fahad Cardiac Center, King Saud University Medical City, King Saud University, Riyadh, Saudi Arabia

**Keywords:** Coronary artery bypass grafting, Minimized cardio-pulmonary bypass, Mini-bypass, Diabetic patients, Morbidity and mortality

## Abstract

**Introduction:**

Multiple studies have shown a decrease in the inflammatory response with minimized bypass circuits leading to less complications and mortality rate. On the other hand, some other studies showed that there is no difference in post-operative outcomes. So, the aim of this study is to investigate the clinical benefits of using the Minimized cardiopulmonary Bypass system in Coronary Artery Bypass Grafting and its effect on postoperative morbidity and mortality in diabetic patients as one of the high-risk groups that may benefit from these systems.

**Methods:**

This is a retrospective study that included 114 diabetic patients who underwent Coronary artery bypass grafting (67 patients with conventional cardiopulmonary bypass system and 47 with Minimized cardiopulmonary bypass system). The patients’ demographics, intra-operative characteristics and postoperative complications were compared between the two groups.

**Results:**

Coronary artery bypass grafting was done on a beating heart less commonly in the conventional cardiopulmonary bypass group (44.78% vs. 63.83%, *p* = 0.045). There was no difference between the two groups in blood loss or transfusion requirements. Four patients in the conventional cardiopulmonary bypass group suffered perioperative myocardial infarction while no one had perioperative myocardial infarction in the Minimized cardiopulmonary bypass group. On the other hand, less patients in the conventional group had postoperative Atrial Fibrillation (4.55% vs. 27.5%, *p* = 0.001). The requirements for Adrenaline and Nor-Adrenaline infusions were more common the conventional group than the Minimized group.

**Conclusion:**

The use of conventional cardiopulmonary bypass for Coronary Artery Bypass Grafting in diabetic patients was associated with higher use of postoperative vasogenic and inotropic support. However, that did not translate into higher complications rate or mortality.

## Introduction

The use of Cardiopulmonary Bypass (CPB) machine has been associated with multiple problems that include hemodilution, complement and white cell activation with systemic inflammatory response, platelet activation, the need for intensive anticoagulation, systemic organ dysfunction, and the frequent need for blood and blood products to control post-bypass bleeding or blood loss [1, 2].

Off-Pump Coronary Artery Bypass Grafting (OPCAB) has been advocated to address some of these concerns [3, 4]. However, OPCAB is currently performed in fewer of 25% of Coronary Artery Bypass Grafting (CABG) procedures [5, 6] because of the technical difficulties encountered in this procedure, the questionable effect on long-term graft patency particularly those of venous grafts [7], and the risk of incomplete revascularization [8, 9]. due to the complex anatomy of coronary lesions.

Alternatively, minimized Cardiopulmonary Bypass (Mini-Bypass) systems have been developed to minimize some of the problems associated with the conventional CPB in order to achieve better outcomes and fewer complications after CABG surgery [10, 11]. The use of these systems has been shown to be associated with less blood loss during the immediate postoperative period [12]. This is of particular importance because the administration of red blood cells can increase postoperative morbidity and mortality. Even after a successful surgical outcome, red blood cell transfusion has also been shown to reduce long-term survival.

Immer and colleagues found also improved myocardial protection in patients undergoing surgery with the mini-bypass system compared to the conventional CBP [13]. Multiple other studies have shown a decrease in the inflammatory response with minimized bypass circuits leading to less myocardial dysfunction, respiratory failure, renal insufficiency, stroke and consequently less mortality than conventional CPB [14–17].

Despite all these potential beneficial effects for the mini-bypass systems, some other studies showed that there is no difference in post-operative outcomes [2, 10, 18–24]. These discrepancies in clinical outcomes between different studies suggest that patients enrolled in those studies may represent a heterogenous group of patients who may benefit especially high-risk patients and those who may not benefit.

Based on these findings, it is important to investigate the effect of these pumps on different high-risk groups in order to find out which group of high-risk patients would benefit from the use of these improved systems. Diabetic patients are one of these high-risk groups that might benefit from these mini-bypass systems. So, the aim of this study is to investigate the clinical benefits of using the Mini-Bypass system in CABG and its effect on postoperative morbidity and mortality in diabetic patients.

## Methods

This is a retrospective study that included 114 diabetic patients who underwent CABG by the same surgeon. Sixty-seven patients underwent CABG with the use of conventional CPB and forty-seven patients with the use of Mini-Bypass. The choice between the two techniques was based on the surgeon’s discretion. Patients who underwent redo operations or combined CABG and other procedures were excluded. The study was approved by the institutional review board (IRB) at the hospital and were conducted in accordance with Good Clinical Practice. Written informed consent was waived by the ethics committee.

Sorin Stockert’s S3 Heart-Lung Machine was used for the conventional CPB control group. The system included a roller pump with a hollow fiber oxygenator with a collapsible venous reservoir. The pump was primed with 1.5 L of Ringer Lactate solution. Other additives included 100 ml of Albumin 25%, 25 g of mannitol, 0.5 g of solumedrol and 5000 IU of Heparin. The Mini-bypass system included a closed circuit composed of centrifugal pump (Medtronic BPX-80 BIO-Pump Plus with a tip to tip coating with Cortiva BioActive surface and non-leaching end point attached heparin coating technology) with a hollow fiber oxygenator with integrated arterial filter and venous bubble trap for managing venous air with no venous reservoir. The Mini-Bypass system was used on the same S-III heart-lung machine. It was primed with 800 ml of Ringer’s lactate with the same additives like the conventional CPB. A retrograde autologous blood priming technique was performed after arterial cannulation and before initiation of CPB in the mini-bypass group only. Negative pressure venous suction was used for venous drainage. Both groups received the same protocol of insulin infusion intra-operatively to maintain normoglycemia in the range of 6–8 mmol/L.

The data was collected using patients’ medical records in addition to cardiac surgery discharge summaries database, anesthesia records, perfusion records and blood bank transfusion records. Continuous variables were compared using either the two-sample *t*-test or the Wilcoxon rank sum test as appropriate by the distribution of data. Categorical variables will be compared using Chi-Square test or Fisher’s exact test depending on the number of items in each group. A p- value of less than 0.05 was considered statistically significant. The Statistical analysis was performed using stata13.1 software.

## Results

A hundred and fourteen patients were included in the study, 67 patients had CABG with the use of conventional CPB and 47 with the use of Mini-Bypass. The conventional CPB group included more hypertensive patients (74.63% vs. 55.32%, *p* = 0.031), more patients with left main coronary disease (17.9% vs. 6.38%, *p* = 0.073) and ejection fraction (EF) was higher in this group (0.52 ± 0.15 vs. 0.44 ± 0.14, *p* = 0.008). The rest of demographic characteristics were similar between the two groups (Table 1).

**Table 1:** Patients’ demographic Characteristics

	CPB *N* = 67(58.77%)	Mini-Bypass *N* = 47(41.23%)	*P*-value
Male	63 (94.03%)	42 (89.36%)	0.363
Smoking History			0.626
Ex-Smoker	13 (19.40%)	9 (19.15%)	
yes	13 (19.40%)	6 (12.77%)	
No	41 (61.19%)	32 (68.09%)	
Chronic Lung Disease	6 (8.96%)	4 (8.51%)	0.934
HTN	50 (74.63%)	26 (55.32%)	0.031
Diabetes Management			0.106
Diet	0 (0.00%)	2 (4.26%)	
Insulin	10 (14.93%)	11 (23.40%)	
Oral Hypoglycaemics	57 (85.07%)	34 (72.34%)	
No of Diseased CA			0.091
1-vessel	1 (1.49%)	0 (0.00%)	
2-vessel	9 (13.43%)	1 (2.27%)	
3-vessel	57 (85.07%)	43 (97.73%)	
Left_Main__50	12 (17.91%)	3 (6.38%)	0.073
MI	26 (38.81%)	11 (23.40%)	0.084
Types of MI			0.271
NSTEMI	19 (73.08%)	6 (54.55%)	
STEMI	7 (26.92%)	5 (45.45%)	
Preoperative Stroke	1 (1.49%)	0 (0.00%)	0.400
NYHA			0.492
Class I	4 (5.97%)	1 (2.13%)	
Class II	25 (37.31%)	15 (31.91%)	
Class III	33 (49.25%)	29 (61.70%)	
Class IV	5 (7.46%)	2 (4.26%)	
AF	2 (2.99%)	0 (0.00%)	0.232
Pre-operative EF	0.52 ± 0.15	0.44 ± 0.14	0.008
Chronic Renal Failure	0 (0.00%)	1 (2.13%)	0.230
Pre-Op Creatinine (μmole/L)	94.52 ± 23.10	97.55 ± 20.11	0.469
Logistic Euro Score	5.97 ± 9.30	5.39 ± 5.80	0.704

HTN: Hypertension, CA: coronary arteries, MI: myocardial infarction, NSTEMI: Non-ST elevation myocardial infarction, STEMI: ST elevation myocardial infarction, NYHA: New York Heart Association, AF: atrial fibrillation, EF: ejection fraction

All patients underwent CABG with the use of left internal mammary artery (LIMA) and vein grafts. The median number of bypasses was 3 bypasses in both groups. The procedure was done on a beating heart less commonly in the conventional CPB group (44.78% vs. 63.83%, *p* = 0.045). There was no difference in the median CPB duration between the two groups (74 ± 55 min vs. 75 ± 43 min, *p* = 0.73).

**Fig. 1:**
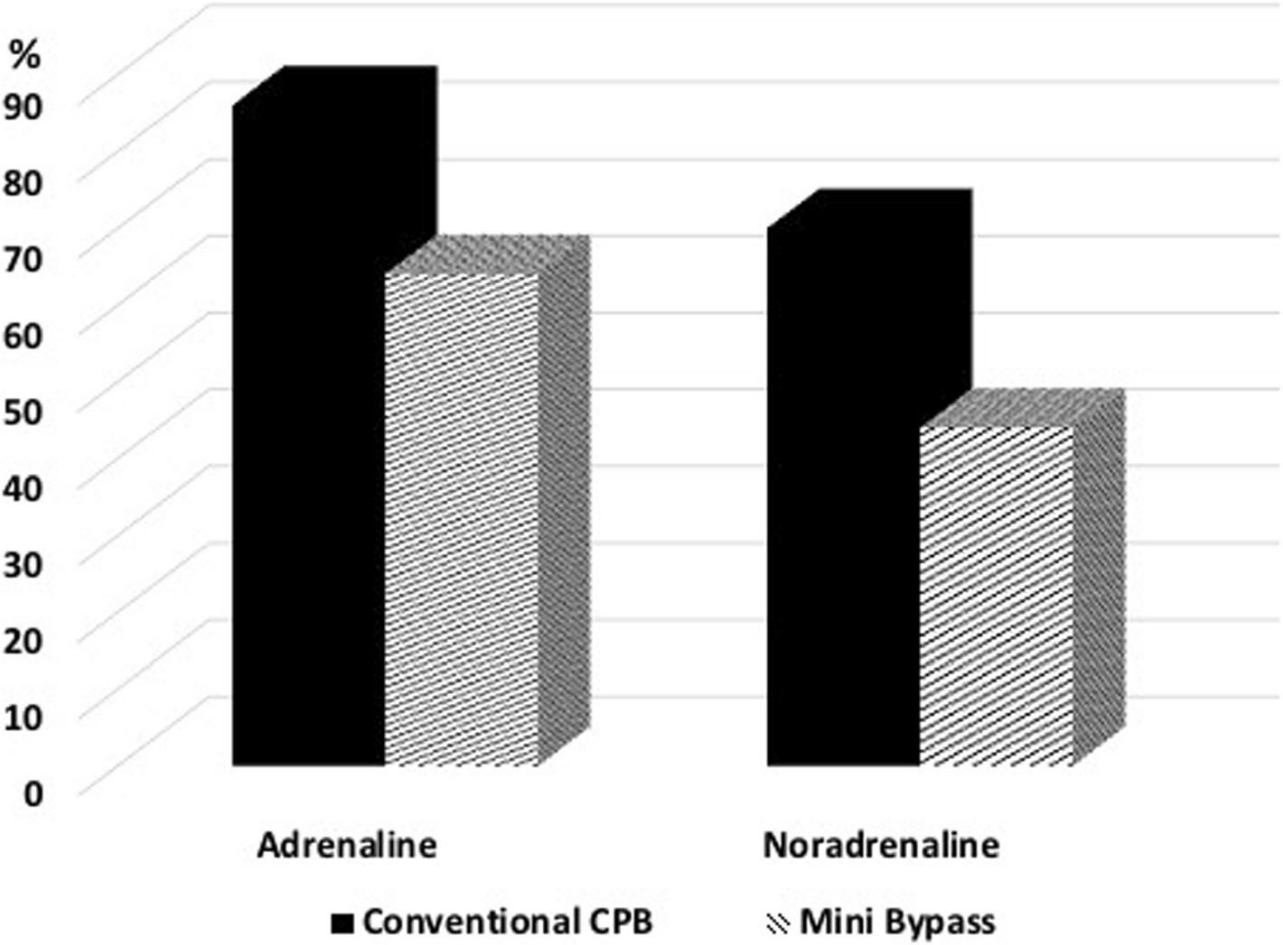
Adrenaline and Nor-Adrenaline Use. illustrates the percentage of patients who required postoperative Adrenaline and Nor-Adrenaline in conventional CPB group (solid black) vs the Mini-Bypass group (dashed lines)

There was no difference between the two groups in blood loss or transfusion requirements. Four patients in the conventional CPB group suffered perioperative myocardial infarction (MI) while no one had perioperative MI in the Mini-Bypass group. On the other hand, less patients in the conventional group had postoperative Atrial Fibrillation (4.55% vs. 27.5%, *p* = 0.001). The requirement for Adrenaline and Nor-Adrenaline infusions were more common the conventional group than the Mini-Bypass group (Fig. 1). The rest of postoperative complications were similar between the two groups as shown in Table 2.

**Table 2:** Postoperative Characteristics and Complications

	CPB N = 67(58.77%)	Mini-Bypass N = 47(41.23%)	P-value
Estimated Blood Loss Post-Op, median (IQR)	1060 (730.0)	1060 (685.0)	0.560
Reoperation for Bleeding	3 (4.48%)	4 (8.51%)	0.377
No of transfused PRBCs, median (IQR)	3.00 (5.00)	3.00 (4.00)	0.862
No of transfused FFPs, median (IQR)	6.00 (8.00)	4.00 (4.00)	0.717
No of transfused platelets, median (IQR)	6.00 (5.00)	6.00 (1.00)	0.421
Mediastinitis	2 (2.99%)	0 (0.00%)	0.232
Leg Wound infection	1 (1.49%)	1 (2.13%)	0.799
Urine Infection	1 (1.49%)	1 (2.13%)	0.799
Any Infection	3 (4.48%)	1 (2.13%)	0.502
Post-Operative Noradrenaline Use (mcg)	44 (69.84%)	20 (43.48%)	0.006
Post-Operative Duration of Noradrenaline Use (minutes), median (IQR)	312.0 (1140)	0.00 (150.0)	0.001
Post-Operative Adrenaline Use (mcg)	53 (85.48%)	30 (63.83%)	0.009
Post-Operative Duration of Adrenaline Use (minutes), median (IQR)	695.0 (958.0)	705.0 (1035)	0.287
Postoperative AF	3 (4.55%)	11 (27.50%)	<.001
Perioperative MI	4 (6.06%)	0 (0.00%)	0.086
Postoperative Stroke	1 (1.49%)	0 (0.00%)	0.400
Postoperative Renal Failure Requiring Dialysis	1 (1.49%)	2 (4.26%)	0.364
Peak Post-Op Creatinine (mmol/L), median (IQR)	124.0 (40.00)	115.0 (38.00)	0.418
Re Intubation	0 (0.00%)	1 (2.13%)	0.230
Pneumonia	2 (2.99%)	0 (0.00%)	0.232
Pre-Op Weight, median (IQR)	73.30 (8.60)	74.00 (19.80)	0.698
Max Post-Op Weight, median (IQR)	75.20 (11.20)	76.00 (20.40)	0.917
Max Post-Op WBC Count, median (IQR)	23.30 (6.60)	20.00 (10.10)	0.019
Lowest Post-Op Albumin Lev, median (IQR)	27.00 (5.00)	27.00 (6.00)	0.827
Amount of Insulin used (units), median (IQR)	274.5 (62.00)	246.0 (107.0)	0.067
Hospital Death	3 (4.48%)	2 (4.26%)	0.955
Cause			
Multi-organ failure	3 (4.48%)	2 (4.26%)	0.955
Length of ICU stay, median (IQR)	48.00 (46.00)	48.00 (12.00)	0.646
Ventricular Tachycardia/ V. Fib	2 (3.03%)	1 (2.13%)	0.769

IQR: Interquartile Range, PRBC: packed red blood cells, FFP: fresh frozen plasma, AF: atrial fibrillation, MI: myocardial infarction, WBC: white blood cells, ICU: intensive care unit

## Discussion

The systemic inflammatory response (SIR) induced by CPB is the result of the activation of both cellular and humoral components. Inflammatory response activation may contribute to myocardial dysfunction, respiratory failure, renal insufficiency, confusion or stroke, atrial fibrillation and coagulopathy [1, 2]. Multiple studies noted a decrease in the inflammatory response with Mini-Bypass circuits [2, 13, 19, 25, 26].

The rationale of miniaturization of extracorporeal circuits is to reduce foreign surfaces as well as priming volume and, therefore, to limit SIR and alterations in perioperative hemostasis. This is achieved through suction blood separation, biocompatible coating, reduction of the total length of the circuit and reduction of prime volume. The mini-bypass system includes an integrated venous bubble trap, centrifugal pump, heat exchanger, and oxygenator and is designed for use with an autotransfusion/cell saving system for sequestration of aspiration blood.

Several studies have shown that in coronary bypass surgery, the mini-bypass system, used as a total CPB, reduces SIRS compared to standard CPB circuit [1, 10, 27, 28]. Whether the reduction of inflammatory response with the use of Mini-Bypass systems would result in improved clinical outcomes is still controversial. A systematic review and meta-analysis were conducted by Zangrillo and colleagues and they showed that the use of Mini-Bypass system results in decreased transfusion rate and cardiac and neurologic damage [29]. On the other hand, another meta-analysis by Winkler and colleagues and other prospective studies showed no difference in clinical outcomes with the use of Mini-Bypass [10, 19, 24, 30].

This heterogeneity in the results of Mini-Bypass pumps illustrates the need to study it use in high-risk patients to tease out its real benefit. We investigated in our study the effects of using the Mini-Bypass pump in diabetic patients and we showed that it decreased the use of vasogenic support postoperatively which can be explained by the initially illustrated studies that it decreases inflammatory response. Additionally, there was less use of Inotropic support postoperatively despite including patients with lower EF in this group that may indicate better myocardial protection. Although some of these findings have been shown previously, this is the first report of these findings in diabetic patients. This has a significant clinical impact since diabetic patients has more extensive vasculopathy and can benefit significantly from reduction of vasogenic drugs postoperatively. Reduction of inotropic support in diabetic patients with low EF also is very important since these hearts are chronically depleted from energy stores and exposing them to high doses of inotropic drugs may further increase their injury and delay their recovery. Although it did not reach statistical significance, the incidence of perioperative MIs showed a trend toward less MI rate in the Mini-Bypass group. This an important signal that need to be studies in the future in this diabetic high-risk group. Multiple pathophysiological explanation could be responsible for this finding including reduction of myocardial edema and the inflammatory response that may improve early graft patency. Another important signal detected in our study was the lower dose of intravenous insulin that was administered intraoperatively in the Mini-Bypass group to maintain normoglycemia, which could be explained by the reduction of insulin resistance as a result of the reduced inflammatory response with the use of these Mini-Bypass pumps.

This study has multiple limitations due to the nature of retrospective studies in addition to its small sample size but it focuses on a specific group of high-risk patients and its sheds the light on important clinical findings that need to be investigated on a higher scale.

## Conclusion

The use of conventional CPB for CABG in diabetic patients was associated with higher use of postoperative vasogenic and inotropic support. However, that did not translate into higher complications rate or mortality. More studies are needed in the future to explore this effect.

## Data Availability

The datasets generated and/or analyzed during the current study are not publicly available due to the institutional patients’ confidentiality policy but are available from the corresponding author on reasonable request.

## Abbreviations

CPB: Cardiopulmonary Bypass
OPCAB: Off-Pump Coronary Artery Bypass Grafting
CABG: Coronary Artery Bypass Grafting
Mini-Bypass: Minimized Cardiopulmonary Bypass
IRB: Institutional Review Board
EF: Ejection Fraction
LIMA: Left Internal Mammary Artery
MI: Myocardial Infarction
SIR: Systemic Inflammatory Response
